# Measuring Poverty in Southern India: A Comparison of Socio-Economic Scales Evaluated against Childhood Stunting

**DOI:** 10.1371/journal.pone.0160706

**Published:** 2016-08-04

**Authors:** Deepthi Kattula, Srinivasan Venugopal, Vasanthakumar Velusamy, Rajiv Sarkar, Victoria Jiang, Mahasampath Gowri S., Ankita Henry, Jordanna Devi Deosaran, Jayaprakash Muliyil, Gagandeep Kang

**Affiliations:** 1 Department of Gastrointestinal Sciences, Christian Medical College, Vellore, Tamil Nadu, India; 2 Department of Biology, Georgia Institute of Technology, Atlanta, Georgia, United States of America; 3 Department of Biostatistics, Christian Medical College, Vellore, Tamil Nadu, India; 4 Department of Biology, Denison University, Granville, Ohio, United States of America; 5 Division of Medical and Dental Education, University of Aberdeen, Aberdeen, Scotland, United Kingdom; Leibniz Institute for Prevention Research and Epidemiology (BIPS), GERMANY

## Abstract

**Introduction:**

Socioeconomic status (SES) scales measure poverty, wealth and economic inequality in a population to guide appropriate economic and public health policies. Measurement of poverty and comparison of material deprivation across nations is a challenge. This study compared four SES scales which have been used locally and internationally and evaluated them against childhood stunting, used as an indicator of chronic deprivation, in urban southern India.

**Methods:**

A door-to-door survey collected information on socio-demographic indicators such as education, occupation, assets, income and living conditions in a semi-urban slum area in Vellore, Tamil Nadu in southern India. A total of 7925 households were categorized by four SES scales—Kuppuswamy scale, Below Poverty Line scale (BPL), the modified Kuppuswamy scale, and the multidimensional poverty index (MDPI) and the level of agreement compared between scales. Logistic regression was used to test the association of SES scales with stunting.

**Findings:**

The Kuppuswamy, BPL, MDPI and modified Kuppuswamy scales classified 7.1%, 1%, 5.5%, and 55.3% of families as low SES respectively, indicating conservative estimation of low SES by the BPL and MDPI scales in comparison with the modified Kuppuswamy scale, which had the highest sensitivity (89%). Children from low SES classified by all scales had higher odds of stunting, but the level of agreement between scales was very poor ranging from 1%-15%.

**Conclusion:**

There is great non-uniformity between existing SES scales and cautious interpretation of SES scales is needed in the context of social, cultural, and economic realities.

## Introduction

In 2005, it was estimated that 1.4 billion people lived in extreme poverty with alarge gap between the rich and the poor[1].Hence one of the Millennium Developmental Goals (MDG) was to reduce world poverty by 15% by 2015.The measurement of poverty or material disadvantage and its comparison at national and international level is challenging. The measurement of socioeconomic status (SES) in the population provides important information for the analysis of inequality and poverty, based on which economic and public health policies and interventions are planned or implemented[2].

SES has been an important predictive variable in epidemiologic and social science studies. People with low SES have low life expectancy, higher mortality, morbidity and under-nutrition [3–6].There are numerous SES scales, designed to capture the social and economic status of populations and groups. Usually, SES is calculated using measurements of different domains including education, occupation, income, material possessions and living conditions or the built environment of the home. All available scales rank or weigh domains differently. A system widely used in India was established by Dandekar and Rath in 1971, to assess per capita income/ consumption expenditure by caloric intake[7], while the Kuppuswamy scale uses education and occupation of the head of the household[8]. The Kuppuswamy scale is widely used for urban populations and was proposed in 1976[8], with income criteria revised periodically based on inflation. Income is a difficult indicator in settings where data is based on self-reporting and respondents under-or over-state income. This was noted in previous studies, and a modified version of Kuppuswamy scale using a five-point scale was created, in which assets and housing structure were considered instead of income[9].The Below Poverty Line (BPL) scale was designed by the Government of India to identify economically disadvantaged households/individuals needing government aid and subsidies. The 10^th^plan BPL survey for urban families was based on the extent of deprivation with respect to seven parameters: roof, floor, water, sanitation, education level, type of employment and status of children in a house[10].The Multi-Dimensional Poverty Index (MDPI) is an international scale developed by the Oxford Poverty and Human Development Initiative and the United Nations Development Programme in 2010[11] to measure acute poverty and designed to identify the most vulnerable people, the poorest among the poor.

Evaluating the SES of households/individuals requires good quality survey data and needs a conceptually coherent and empirically robust scale to capture the economic conditions of households in the context of resource availability and access in their environment. In order to have a better understanding of measures of poverty or of socio-economic status, it is important to explore different systems of classification in the same population and to validate the classification with objective measures. This study compared indicators and categorization of a population using four SES scales, Kuppuswamy, BPL, modified Kuppuswamy and the MDPI in order to assess agreement on categorization of low SES between scales and to estimate the association of SES scales with stunting in children as an indicator because stunting is considered as the best surrogate marker for child health inequalities and long term deprivation[12–14].

## Material and Methods

### Study area and population

**Fig 1:**
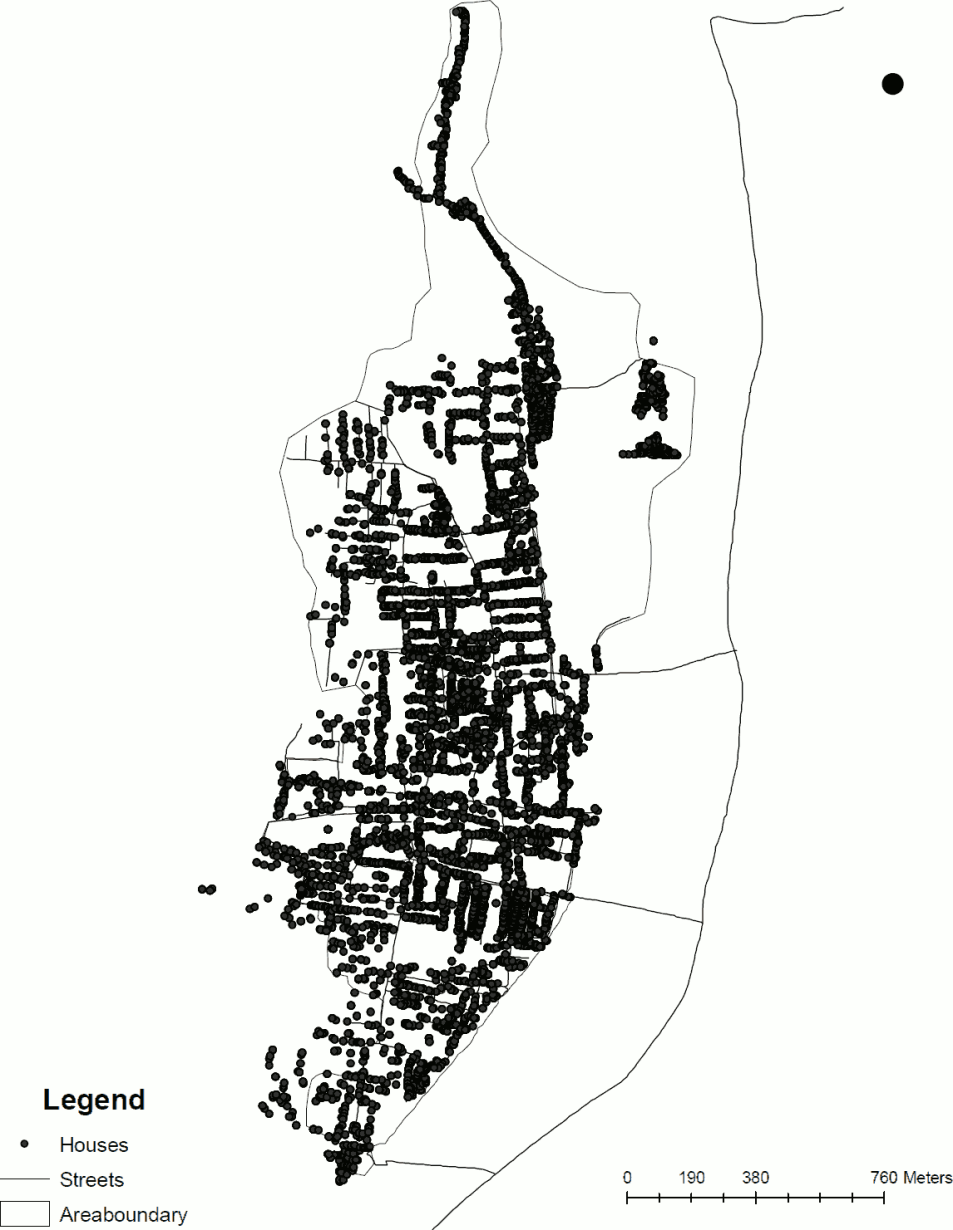
Geographic distribution of study population.

A population based survey was conducted in four geographically adjacent, semi-urban slums[15], Ramnaickapalayam, Chinnallapuram, Kaspa and Vasanthapuram, located in the western outskirts of Vellore, Tamil Nadu in south India covering an area of 2.2 Km^2^ and with an approximate population of 43000 individuals (Fig 1). The Urban Health Centre for the area documented a birth rate of 15.3 live births per 1000 population per year and an infant mortality rate of 18.2 deaths per 1000 live births per year for the years 2008–2011. The sample registration survey during the same period conducted by the Government of India reported birth and infant mortality rates for urban Tamil Nadu as 15.8 live births per 1000 population and 22 deaths per 1000 live births, respectively[16].

#### Data collection

A door-to-door survey was conducted by 30 field workers from September to November 2010. In case of locked doors, field workers re-visited the houses till information was obtained. A total of 8028 households were surveyed and complete information was obtained for 7925 households. The survey questionnaire covered various socio-demographic domains, with major indicators including education, occupation, living conditions and possessions (S1 Appendix). In addition, anthropometric measurements of length/height (cm) and weight (kg) were taken from the households with children less than 5years of age at the time of the survey by using calibrated infantometer or stadiometer and electronic weighing machines respectively. Field workers were trained in data collection and in anthropometric measurement in order to standardize data collection. We measured height and weight for all the under 5 children in a household. If children were absent, the household was revisited until we obtained measurements from the children who were not present at the time of survey. Weekly validation of 10% of all data was conducted by the field supervisor as a quality check. All census forms were checked by the data managers for completeness and errors and sent back to the field for corrections.

The decision maker in the family was considered as the head of the household and the highest earner in the family was considered the primary earner of the household. A family was termed a joint family if two or more married siblings/cousins lived in the same household with their respective nuclear family members and had a common kitchen. In ‘extended’families grandparents lived with nuclear families, i.e., people from three generations living in the same household. Types of houses were categorized by the type of roofs and floors: a hut or a house with mud walls with thatched roof was a “kutcha” house, whereas a house with a concrete roof, floor and walls was a “pucca” or permanent house. Mixed houses had components of kutcha and pucca houses. The type of indicators used for the computation of the SES scores varied between scales and are shown in Table 1.

**Table 1:** Indicator variables used in the Kuppuswamy, Below Poverty Line, modified Kuppuswamy scales and the Multi-Dimensional Poverty Index scales.

Indicators	Kuppuswamy scale	Below Poverty Line scale	Modified version Kuppuswamy scale	Multi-Dimensional Poverty Index scale
**Education**	Education of the head of the household	Education of the primary earner of the household	Highest education in the household has more than 11 yrs of education	Adults with no primary education in the household
**Occupation**	Head of household	Primary earner of the household	Head of the household	Not included
**Income**	Family income per month	Not included	Not included	Not included
**Living conditions/built environment**	Not included	Type of floor, roof, water, sanitation	Ownership, number of living rooms, assets of the household, cooking mode, place of cooking	Electricity, sanitation, water, floor, cooking fuel, assets
**Status of child in the family**	Not included	Any school aged child not attending school	Not included	Any school aged child not attending school
**Health status**	Not included	Not included	Not included	Child mortality, malnutrition (LAZ/HAZ < -2SD)

The data collection was conducted as part of the annual census update of the study population which forms the Vellore urban demographic surveillance area. Verbal consent was taken from parents or guardians for measurement of height and weight of children and documented on study forms, unless the children were part of other studies, for which written informed consent was obtained separately. The conduct of the census and anthropometry was approved by the Institutional Review Board of the Christian Medical College, Vellore.

### Statistical Analysis

The survey forms were scanned using an optical character recognizing (OCR) machine and data were analyzed using STATA 10.1 for Windows (Stata Corp, College Station, TX, USA) software. The survey indicators were used to calculate SES as defined by the Kuppuswamy scale[17], the BPL scale used by Government of India in its welfare programmes, the modified Kuppuswamy scale[9, 18] and theMDPI[11].The Kuppuswamy scale categorizes 5 groups, namely lower, upper lower, middle, lower upper, upper. The BPL Scale categorizes the population into 5 different priority classes, I-V. The modified Kuppuswamyscale groups the population into 3 classes, low, middle and high. The MDPI classifies the population into 2 groups, poor or not in multiple dimensions.

Latent class analysis was performed to estimate the sensitivity and specificity of SES scales as there is no specific gold standard for comparison. For each scale, the lowest category was considered ‘low’ (lower in Kuppuswamy scale, priority I in BPL scale, low in modified version of Kuppuswamy scale and multi-dimensionally deprived in MPDI), while the rest of the categories were combined and were considered ‘non low’ for the analysis.Low and non-low SES by the four scales were included in the model and a gold standard latent variable for these two categories. Based on the marginal and conditional probabilities obtained from the model, the prevalence of low SES, sensitivity and specificity were predicted. The magnitude of the association of SES with childhood stunting was estimated using logistic regression for each scale. The height-for-age/length-for-age (HAZ/LAZ) for children was calculated using 2006 WHO child growth standards as the reference population[19] and any child with HAZ/LAZ<-2 SD was considered stunted.

The association of SES scales with stunting was confined to the subgroup of households with ≤ 5year old children. Data from only child per household was taken i.e., if there were two or more children <5 years in a household, the youngest was included for analysis. The results of the association are presented as odds ratio with 95% confidence intervals (OR, 95% CI).With the BPL scale, there were very few households in Priority I, so Priority II was also included for analysis. For analysis of association with stunting with MDPI, a modified MDPI score was obtained by excluding stunting, because stunting is included in the calculation of MDPI scale and the exposure and outcome would be correlated. A test of significance was performed to analyze the trend of stunting among all the SES scales using Chi square for trend analysis [20].Agreement of low socioeconomic status between different scales was assessed using Cohen’s Kappa statistic test[21].

## Results

### Demographic description of study population

The population of 37317 in was 49% males and 51% females. About 9% of the population was children aged <5 years, and 29% of households had at least one child less than 5 years old. The median (IQR) number of children (<12yrs) per household was 2 (1–2).Approximately three-quarters(76.6%) of the population lived in nuclear families with an average (SD) family size of 5(2) members per household. About 53.1% were Hindus and 41.8% of people were Muslims, and the remainder Christians. Among the primary earners, 20.6% were illiterate and 45.1% had high school education. The proportion of households in the community where the head was illiterate was 32.7%. The majority of the primary earners (58.6%) had a semi-skilled/skilled occupation; whereas a large percent of the heads of the households (68.73%) were either unemployed or engaged in unskilled labour. Only 1.4% of heads of households held professional employment, such as engineers, lawyer, nurses, and government officials. Twenty percent of households were headed by women. Approximately 60% of the families in the area earned an income ≤5000 (USD 83, 1USD = Rs.60) per month and only 2% of the households had a family income more than Rs.20000 (333 USD) per month.

Sixty three percent of the families resided in ‘pucca’or permanent houses and 10.8% had ‘kutcha’ houses. Approximately 3% of families lived in a house with an earthern or unplastered floor. Most houses (84.2%) had less than 3 rooms, excluding kitchens and bathrooms and 61.5% of families owned their home. Over two-thirds (70.3%) of households had private pour flush latrines, but 20% of the population defecated in the open and 10% used public pour flush latrines, respectively. The predominant source of water was the public pump and taps supplied by the Vellore municipal corporation (60%) and the remaining 37% had private taps, pumps and bore well in their households. Of school going age children (6–12 years), 85% attended school regularly. The baseline demography and description of the living conditions of the population are presented in Table 2.

**Table 2:** Socio- demographic description of the study population.

Indicator	Number of Households (%)	Indicator	Number of Households (%)
**Type of family**			
Joint	795 (10.03)	**Occupation of primary earner**	
Extended	1056 (13.32)	Unemployed/ Unskilled laborer	1229 (15.51)
Nuclear	6074 (76.64)	Semi skilled/Skilled	4642 (58.57)
		Self employed/ street vendor/push cart driver	1198 (15.12)
**Religion**		Organized sector	856 (10.80)
Hindu	4209(53.11)		
Christian	406 (5.12)	**Occupation of head of the household**	
Muslim	3310(41.77)	Unemployed/ Unskilled laborer	5447 (68.73)
		Semi skilled/Skilled	1505 (18.99)
**Education of primary earner**		Clerical/shop owner/ landowner/ semiprofessional	863 (10.89
Illiterate	1636 (20.64)	Profession	110 (1.39)
Primary	1473(18.59)		
High School	3574 (45.10)	**Monthly Family income (Rupees)**	
Higher secondary	554 (6.99)	< = 1000	205 (2.59)
Graduate	688 (8.68)	1001–5000	4400 (55.52)
		5001–10000	2388 (30.13)
**Education of head of household**		10001–20000	732 (9.24)
Illiterate	2594 (32.73)	> 20000	200 (2.52)
Primary school	1488 (18.78)		
High School	3052 (38.51)	**Average (SD) family size**	5 (2)
Higher sec/ intermediate	409 (5.16)		
Graduate/ PG/ Professional	382 (4.82)		
**Indicator**	**Number of Households (%)**	**Indicator**	**Number of Households (%)**
**Standard of Living**			
**Type of house**		**Sanitation**	
Pucca	5003(63.13)	Open defecation	1525 (19.24)
Mixed	2067 (16.08)	Public/ community pour flush	828 (10.45)
Kutcha	855 (10.79)	Private pour flush latrine	5572 (70.31)
**House ownership**		**Water**	
Own	4874 (61.50)	No water supply within 500 yards/open well/tank	108 (1.36)
Rented/leased/Government built	3051 (35.5)	Public hand pump/tube well/bore well/public tap	4882 (61.60)
		Private hand pump/tube well/bore well	2512 (31.70)
**Rooms in the household**		Private piped water supply	423 (5.34)
Number of rooms > = 3	1256 (15.85)		
Number of rooms < 3	6669(84.15)	**Status of children(N = 5400)**	
		Working children & not attending school/irregular	811 (15.02)
**Roof**		Children not working & attending school regularly	4589 (84.98)
Thatch/grass/Tarpaulin/Wooden	908 (11.46)		
Asbestos/Tiled	2119 (26.74)	**Possession/Assets**	
Cement	4898(61.80)	Fan	7630 (96.28)
		Tape/recorder/two-in-one	1243 (15.68)
**Floor**		Color/ black & white television	5431 (68.52)
Earthen/Bajri/Bricks	281 (3.55)	Non motorized /Motorized/Both Vehicles	5827 (73.52)
Cement	6716 (84.74)	Steel almaera	6233(78.65)
Chips/tiles	803 (10.13)	Landline/ Mobile/ both phone	5516 (69.60)
Marble	125(1.58)		

### Classification of socioeconomic status by four SES scales

The classification of the study population using the Kuppuswamy, BPL, modified Kuppuswamy scale and MDPI scalesis shown in Table 3. The Kuppuswamy scale classified 7.1% of the population as lower SES and the majority (72.7%) as in the upper lower socioeconomic stratum. According to the BPL scale, only 1% of the families were categorized as priority I, a category considered as the highest priority for government schemes. The MDPI scale classified 5.5% of families as multi-dimensionally deprived. In contrast to all the other SES scales, the modified Kuppuswamy scale classified almost 55.28% of the families as low SES.

**Table 3:** Socio-economic status classification of study families by the Kuppuswamy, Below Poverty Line, Modified Kuppuswamy and Multi-Dimensional Poverty Index scales.

Socio-economic status Classification	Number of families
**Kuppuswamy scale**	
Lower	560 (7.07%)
Upper lower	5763(72.72%)
Lower Middle	1219 (15.38%)
Upper Middle	360 (4.54%)
Upper	23 (0.29%)
**Below poverty line scale**	
I priority(Highest priority)	71 (0.90%)
II priority	738 (9.31%)
III priority	2623 (33.10%)
IV priority	3549(44.78%)
V priority (Lowest priority)	944 (11.91%)
**Modified version of Kuppuswamy scale**	
Low	4381 (55.28%)
Middle	2796 (35.28%)
High	748 (9.44%)
**Multidimensional poverty index**	
Household deprived multi dimensionally	432 (5.45%)
Household without multi dimensional deprivation	7493 (94.55%)

The Kuppuswamy scale and the modified Kuppuswamy scale ranked 0.3% and 9.4% of the population as upper and high SES strata respectively, whereas the BPL and MDPI scale classified approximately 12% and 94.5% of the population as lowest priority and non-deprived families. These results reflect the conservative estimation of low SES and a liberal estimation of high SES by the BPL and MDPI scales.

### Association of socioeconomic scales with childhood stunting

**Fig 2:**
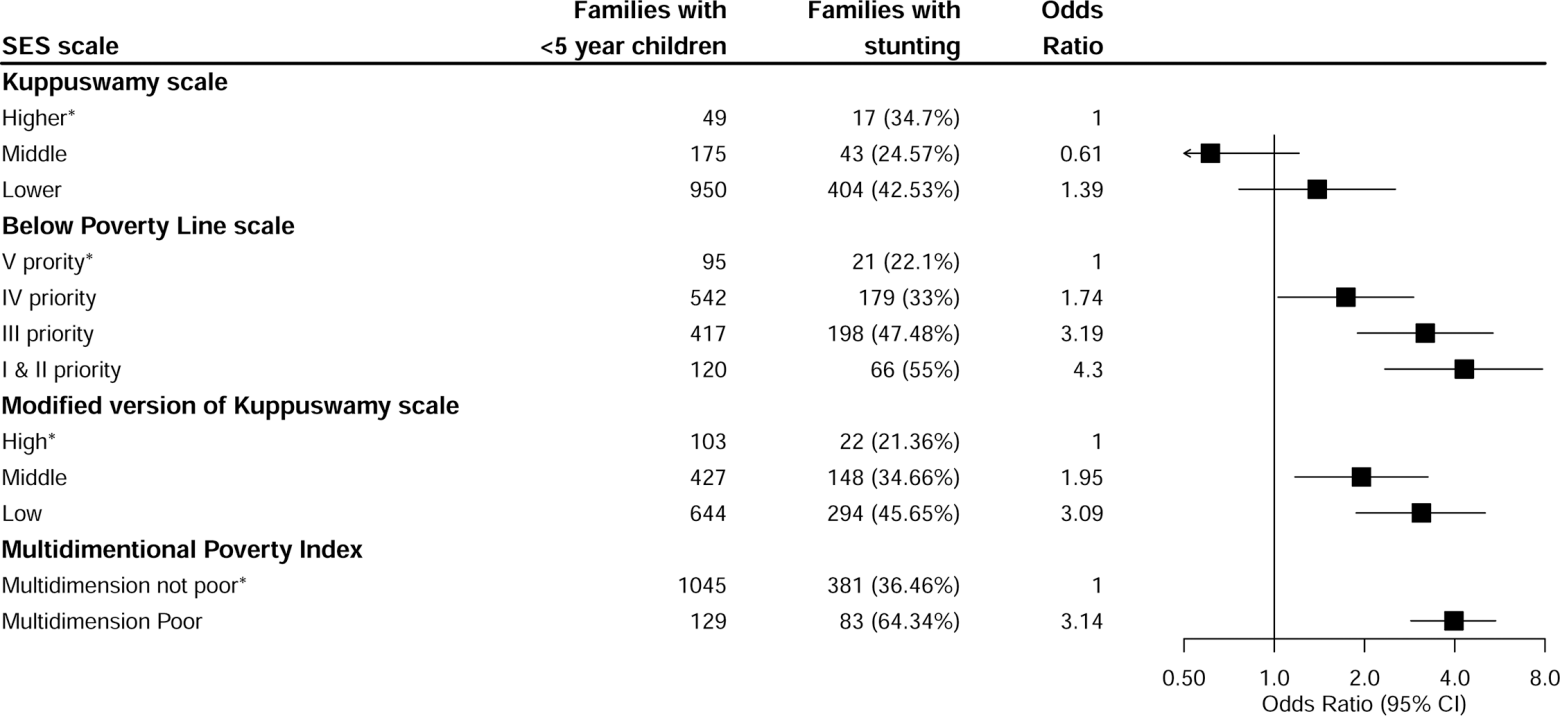
Validation of socio-economic status assessment with stunting in children under the age of five years.

The scales demonstrated significant association between SES and stunting. The proportion of stunted children in the low SES group as classified by the Kuppuswamy, BPL, modified Kuppuswamy and MDPI scales was 42.5%, 75%, 45.7%, and 67% respectively, which was significantly more than the number of stunted children in the high SES group as shown in Fig 2. Logistic regression demonstrated the increase in the odds ratio with a decrease in the order of SES groups, indicating an inverse relation between SES and stunting. By the BPL classification, children from low SEShouseholds(priority I and II)had 4 times (4.34; 2.35–7.87) higher odds of being chronically malnourished, whereas they had three times greater odds by the modified Kuppuswamy scale (3.09; 1.88–5.07) and the modified MDPI (3.14; 2.15–4.61). The Kuppuswamy scale also reported an increased odds of stunting in the low SES(1.32; 0.76–2.54)households, but this was not statistically significant. The test of significance for trend in stunting among all socioeconomic scales was statistically significant (p <0.0001), indicating that with decrease in SES there was increase in stunting. The results are presented in Fig 2.

### Sensitivity and specificity of socioeconomic scales

**Fig 3:**
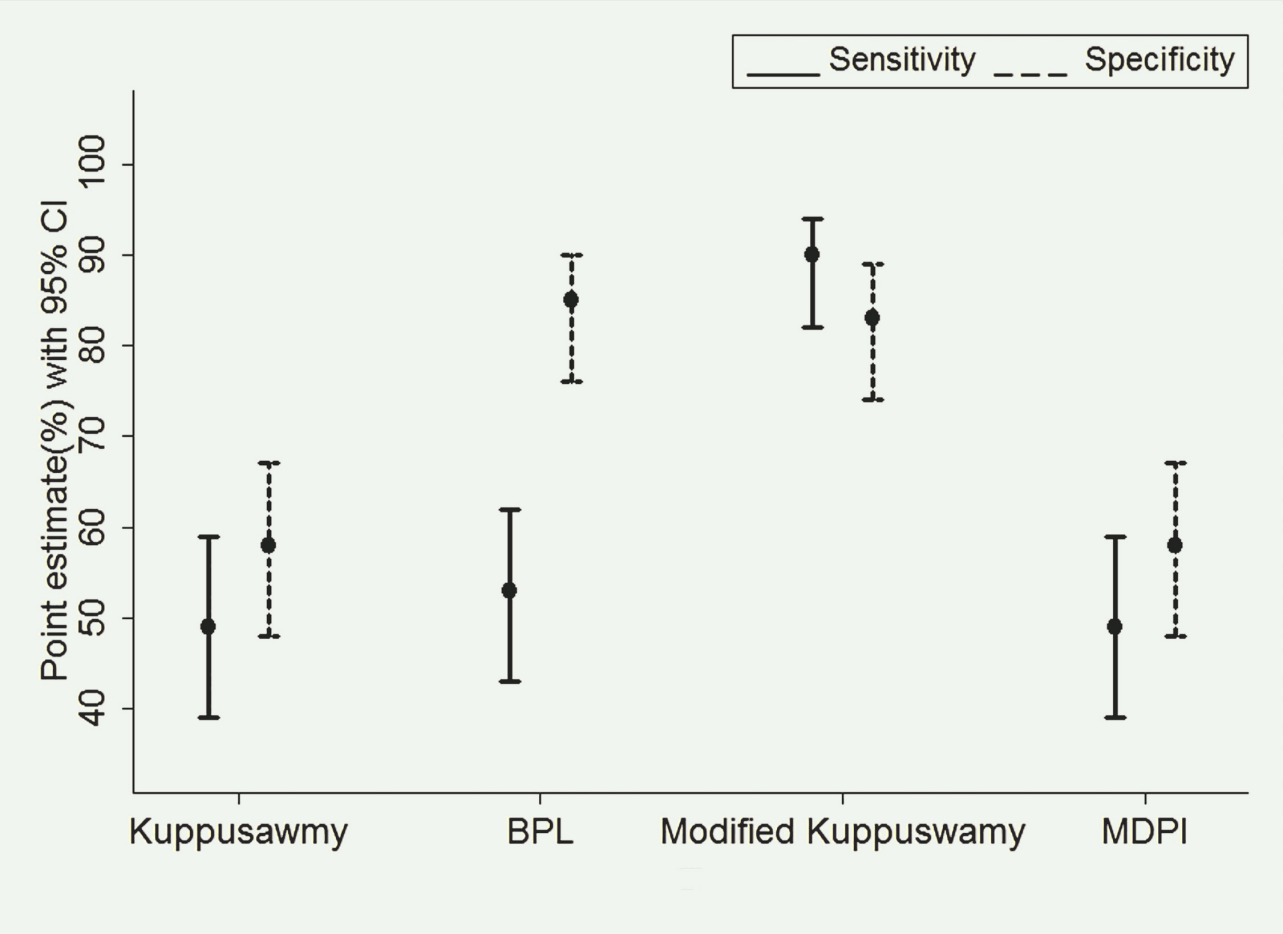
Sensitivity and Specificity for Socio-economic scales.

The modified Kuppuswamy scale had the highest sensitivity (89%) and specificity (83%) to classify poor households in the community compared to the other scales. BPL, Kuppuswamy, MDPI scales had sensitivity approximately around 50%. The BPL scale had the highest specificity of 85% (Fig 3).

Table 4 shows the different agreement between the scales with kappa ranging from 0.01–0.15 of agreement.

**Table 4:** Agreement (Kappa) between four indices.

SES indices	Kuppuswamy		BPL		Modified Kuppuswamy		MDPI	
	Kappa	95% CI	Kappa	95% CI	Kappa	95% CI	Kappa	95% CI
**Kuppuswamy**	1							
**BPL**	0.10	0.08–0.11	1					
**Modified Kuppuswamy**	0.07	0.06–0.08	0.01	0.008–0.01	1			
**MDPI**	0.15	0.13–0.17	0.15	0.13–0.16	0.06	0.07–0.05	1	

## Discussion

This study compared four SES scales, three of which are widely reported and used in India and the fourth is a recently described poverty index derived from international data from several developing countries. This study demonstrates that the difference in emphasis on the attributes measured in each scale results in non-uniform ranking of the same households.

Among the four scales, BPL and MDPI had conservative estimates of the proportion of poor households in an urban semi-organized settlement. Interestingly, a small study from Bangalore, which surveyed 120 households with the Standard living index scale also used in the National Family Health Survey2, which has 11 components; house type, source of lighting, toilet facility, main fuel for cooking, source of drinking water, separate room for cooking, ownership of the house, ownership of agricultural land, ownership of irrigated land, ownership of livestock, ownership of durable goods, also demonstrated a very low (<1%) proportion of poor households[22].

In spite of different sensitivities of the four SES scales, association of the scales with childhood stunting showed strong association with the SES gradient. The magnitude of association with stunting was highest in the BPL scale, although it had a lower sensitivity of just 53%, and classified only 1% as those in need of governmental support; pointing to the fact this scale measures the worst-off households. The modified Kuppuswamy scale had the highest sensitivity (89%), specificity (83%) and good association with stunting. Although Kuppuswamy scale and MDPI had lower sensitivity, these scales also predicted stunting (Fig 2).

Interestingly, the level of agreement between the scales (ranging from 1%-15%) was very poor. Only 30 (0.38%) families were classified as poor by all the four scales. The modified Kuppuswamy scale classified 4381 families as poor, whereas only 459 (10%), 70 (1.6%) and 386 (8.8%) families among them were categorized as poor by the Kuppuswamy, BPL and MDPI respectively. Of the 71 families categorized as the poorest of the poor by the highly conservative BPL scale, almost all families (99%) were also classified poor by the modified Kuppuswamy scale, where as the Kuppuswamy and MDPI scales identified only 35 (49%) and 42 (59%) families as poor, respectively. This variability in the classification of the poor within a defined population, about whom information is available for categorization by multiple scales, raises the question of how much mismatch would result from poverty indices which are used in different populations on a global stage.

As shown in Table 1, most SES scales are primarily based on education, occupation and income, but each scale evaluates these areas differently. Education was considered in all scales but the modified Kuppuswamy scale looked at highest education attained by any member of the family, the Kuppuswamy scale measured education of the head of the household and the BPL scale looked at education of the primary earner in the household. For occupation, the Kuppuswamy and modified Kuppuswamy scales recorded the occupation of the head of the household, whereas BPL took into account the occupation of the primary earner and MDPI did not consider occupation as a variable. Differences in classification by scales could also arise when the head of household is different from the primary earner in the family. Households with senior citizens, who are usually unemployed and get only a small old age pension, as the head of household would fall into a lower category in comparison to the households where the primary earner is the head of the household.

The strengths of the study reported here are the large number of households for whom detailed information could be obtained because of the intensive data collection to assess households by multiple SES scales, unlike most national level surveys and the inclusion of all households, thus avoiding a sampling bias. The weaknesses include the limited geographic region, since variability might be greater with wider sampling. Secondly, whether stunting of children in the household is an appropriate objective measure of chronic deprivation is a matter for consideration, given that there are few other objective measures beyond income.

Two popular SES scales, by Prasad[23]and Kuppuswamy[17],use income as an indicator of SES. Although incomes reflect the purchasing capacity of a family, it can be inconsistent and unreliable, because it is usually under- or over-reported for different reasons. It is also very difficult to measure family income when salaries are not fixed and vary from month to month among daily-wage laborers in non-organized sectors. Dandekar and Rath’s classification was based not just on income but considered level of expenditure per capita per month on all goods and services and daily calorie consumption. According to this classification, which depends on the correlation between the total daily expenditure per person and daily caloric intake, those who do not obtain the standard daily intake of calories (2,400 kilocalories in rural areas and 2,100 kilocalories in urban areas) are below the poverty line[7]. However this calorie intake has not been revised over 35 years, and the government's provision of carbohydrates in various food subsidy programmes, decreases the ability to estimate poverty based on caloric intake.

Living conditions were assessed in the modified Kuppuswamy, BPL and MDPI scales. Certain facilities/amenities such as type of house, water and sanitation, certain possessions/assets such as television, and bicycles are provided by governmental programmes, particularly in Tamil Nadu. Therefore, when scales use presence/availability of such conditions to classify families as non-deprived, they are inaccurate and do not truly reflect the monetary capacity of these families.

Malnutrition and mortality in children less than 5 years have been associated with poverty in developing countries [24–25]. Malnutrition could be due to inadequate nutrition and can also be due to recurrent illnesses in childhood [26]. Both of these are related to poverty. However, only MDPI takes childhood mortality and morbidity into assessment of SES. There is another relationship between economic status and health. Although provision of health service is a core function of health systems, in countries like India where high quality medical care provided by the government is inadequate and difficult to access and health insurance limited; out of pocket expenses can be catastrophic and push people into a lower SES.

## Conclusion

The measurement of socio-economic status and the classification of poverty are critical to understand the conditions in which people live, analyze factors determining this situation, design interventions and monitor and evaluate the effect of interventions aimed at improving living conditions or alleviating poverty. Three crucial aspects must be considered, availability of resources to meet needs as defined by a specified threshold, which may relate to income or consumption, inequality in distribution of an attribute in a population, and vulnerability or the risk of being or becoming poor in the future. Given the variability of the classification by scales used in India, there is clearly an urgent need for standardization.

## Supporting Information

S1 Appendix A survey questionnaire with various socio-demographic domains (PDF)
