# Supplementary material for: Measuring Poverty in Southern India: A Comparison of Socio-Economic Scales Evaluated against Childhood Stunting
Source: PLoS One. 2016 Aug 4;11(8):e0160706. doi: 10.1371/journal.pone.0160706 (PMC4973914; doi:10.1371/journal.pone.0160706)
Supplement: S1 Appendix — (PDF) [file pone.0160706.s001.pdf]

ENUMERATION PROFORMA

Date:

Interviewer **1.1 Fam. ID** **Waypoint No.**

1.2 Head of the household

1.3 D. No/St:

Place: **Pincode**:

1.4 Respondent:

1.5 Religion: 1- Hindu 2-Christian 3- Muslim 4- Others **2.1 No. of members in the household**: **2.2 Adult**: **2.3 Children** (< or = 12 Yrs):

2.4 Type of family: 1- Joint 2- Extended 3- Nuclear

List the Members of the Household

ID No. Name:

Yr. of Birth: Occupation: Marital Status Relation to the Head Education SEX

ID No. Name:

Yr. of Birth: Occupation: Marital Status Relation to the Head Education SEX

ID No. Name:

Yr. of Birth: Occupation: Marital Status Relation to the Head Education SEX

ID No. Name:

Yr. of Birth: Occupation: Marital Status Relation to the Head Education SEX

ID No. Name:

Yr. of Birth: Occupation: Marital Status Relation to the Head Education SEX

ID No. Name:

Yr. of Birth: Occupation: Marital Status Relation to the Head Education SEX

Marital Status: 1- Single 2- Married 3-Widowed 4- Divorced or Separated
Relation to Head: 0-Head, 1-Wife, 2-Son, 3-Daughter, 4-D-in-law, 5-Grandson, 6-Granddaughter, 7-Father, 8- Mother, 9-Brother, 10-Sister, 11-Uncle, 12-Aunt, 13-Nephew, 14-Neice, 15-Mother-in-law, 16-Father-in-law, 17-Son-in-law, 18-Others.

HOUSEHOLD DETAILS – CONTD.

Socio economic factors:

3.1 Type of house: ☐ 1- Pucca 2- Mixed 3- Kutcha

3.2 No. of rooms in the house (excluding kitchen & bathroom): ☐☐

3.3 House ownership: ☐ 1- Own 2- Rented/leased 3-Govt. built

3.4 Possessions:

1. Vehicle	☐	0- No 1- Non-motorised 2- Motorised 3- Both
2. Phone	☐	0- No 1- Land Phone 2- Mobile Phone 3- Both
3. Fridge	☐	0- No 1- Single door 2- double door
4. Washing Machine	☐	0- No 1- Semi automatic 2- automatic
5. Invertor	☐	0- No 1- Yes (how many connections - capacity)

3.5.1 Cooking mode: ☐ 1- Firewood 2- Kerosene Stove 3- Gas Stove 4- More than one

3.5.2 Place of cooking: ☐ 1- Outside the House 2- Inside the House 3- Separate Kitchen

4. Where do you usually go for treatment of minor illnesses? ☐ 1-GH, 2-CMC, 3-CHAD, 4-Private Practitioners, 5- Traditional, 6-Medical Shop 7- UHC, 8-Karigiri Clinic, 9-Ida Scudder Ward [Eye Hospital]

5. Living Condition:

5.1. Roof ☐

1-Thatch/grass 2-Tarpaulin 3-Wooden 4-Asbestos 5-Tiled 6-Cement

5.2. Floor ☐

1-Earthern 2-Bajri (Semi-earthen) 3-Bricks 4-Cement 5-Chips/tiles 6-Marble

5.3. Water ☐

1-No water supply within 500 yards 2-Open well/tank/pond/river 3-Public hand pump/tube well/bore well 4-Public tap 5-Private hand pump/tube well/bore well 6-Private piped water supply

5.4. Sanitation ☐

1-Open defecation 2-Public dry latrine 3-Public/community pour flush latrine 4-Private dry latrine 5-Private pour flush latrine 6-Private flush latrine with sewer connection

6. Education level: ☐ **(for primary earning member)**

1-Illiterate 2-Primary 3-Middle 4-High School (10th Pass) 5-Higher Secondary (10+2 Pass) 6-Graduate

7. Type of employment: ☐ **(for primary earning member)**

1-Unskilled casual laborer/unemployed 2-Semi-skilled 3-Self-employed street vendor/push cart driver 4-Own work place 5-Own work place & selling place

6-Organised sector 7-Others, specify: ______

8.1. Average monthly income of the family: ☐☐☐☐☐☐☐

8.2 Is the income of the primary earning member from daily wages? ☐

0-No 1-Yes

9. Status of children in a household: ☐

1-Working children & not attending any school/ Literacy classes 2-Working children but attending school/ Literacy classes sometimes 3-Working children but attending school/ Literacy classes regularly 4-Children not working as well as not attending any classes 5-Children not working & attending Literacy classes regularly 6-Children not working & attending school regularly
